# Selective Changes in Complexity of Visual Scanning for Social Stimuli in Infancy

**DOI:** 10.3389/fpsyg.2021.705600

**Published:** 2021-11-02

**Authors:** Przemysław Tomalski, David López Pérez, Alicja Radkowska, Anna Malinowska-Korczak

**Affiliations:** ^1^Institute of Psychology, Polish Academy of Sciences, Warsaw, Poland; ^2^Faculty of Psychology, University of Warsaw, Warsaw, Poland

**Keywords:** infancy age, eye-tracking, visual scanning behavior, RQA analysis, social stimuli, complexity

## Abstract

In the 1st year of life, infants gradually gain the ability to control their eye movements and explore visual scenes, which support their learning and emerging cognitive skills. These gains include domain-general skills such as rapid orienting or attention disengagement as well as domain-specific ones such as increased sensitivity to social stimuli. However, it remains unknown whether these developmental changes in what infants fixate and for how long in naturalistic scenes lead to the emergence of more complex, repeated sequences of fixations, especially when viewing human figures and faces, and whether these changes are related to improvements in domain-general attentional skills. Here we tested longitudinally the developmental changes in the complexity of fixation sequences at 5.5 and 11 months of age using Recurrence Quantification Analysis. We measured changes in how fixations recur in the same location and changes in the patterns (repeated sequences) of fixations in social and non-social scenes that were either static or dynamic. We found more complex patterns (i.e., repeated and longer sequences) of fixations in social than non-social scenes, both static and dynamic. There was also an age-related increase in the length of repeated fixation sequences only for social static scenes, which was independent of individual differences in orienting and attention disengagement. Our results can be interpreted as evidence for fine-tuning of infants' visual scanning skills. They selectively produce longer and more complex sequences of fixations on faces and bodies before reaching the end of the 1st year of life.

## Research Highlights

- Using dynamic measures of visual scanning in infants we found more systematic scanning patterns for visual social stimuli, both static and dynamic, in comparison to non-social ones.
- Between 5.5 and 11 months of age there were changes in the length of fixation sequences in visual scenes with people in comparison to those without them.
- Developmental changes in visual scanning patterns for static social stimuli were not related to independently measured oculomotor skills.

## Introduction

Efficient scanning of visual scenes serves as the primary source of information about the surrounding environment in typical development. It allows us to rapidly identify threats, find food or spatial locations of important people and objects. Visual scanning is available to newborns from the 1st min after birth and improves rapidly throughout infancy (see Ruff and Rothbart, 1996). These improvements are partly driven by the development of brain mechanisms controlling voluntary eye movements and attention (for a review see Hendry et al., 2019). By 5–6 months of age infants use spatial location of objects as attention cues (Hood, 1993; Johnson and Tucker, 1996), track moving objects and anticipate their trajectory (Haith et al., 1988). They are also able to inhibit automatic saccades (Johnson, 1995; Holmboe et al., 2008) and to disengage from attractive stimuli at will (Johnson et al., 1991; Holmboe et al., 2018), thus demonstrating improvements in endogenous orienting (Hitzert et al., 2015). As infants develop and their oculomotor system matures, they make more accurate and efficient eye movements, and their scanning patterns change (Hunnius and Geuze, 2004). Because of increasing endogenous control their scanning patterns become more complex when viewing naturalistic scenes (e.g., Kelly et al., 2019). Thanks to these emerging skills (amongst others) their visual scanning is gradually less driven by exogenous cues and more by curiosity, existing knowledge and expectations.

Apart from domain-general improvements in oculomotor control infants also show domain-specific developmental increases in sensitivity to certain classes of objects, especially to social stimuli. These include increased and preferential fixating on faces, eyes, hands and human bodies (Frank et al., 2012), improvements in the scanning consistency and learning to prioritize top-down features like faces (Franchak et al., 2016) as well as more advanced perceptual discrimination of those kinds of faces that are common in an infant's natural environment (e.g., Anzures et al., 2012). This last phenomenon, known as perceptual narrowing, is thought to reflect the specialization of perceptual systems for stimuli that carry important, socially-relevant information. Perceptual narrowing is reflected in increased discrimination of own-species and own-race faces, as well as in changes in the scanning patterns of internal facial features, e.g., increased looking at the eyes of own-race faces only (Wheeler et al., 2011; Wilcox et al., 2013).

To date, the majority of work on infant visual scanning has focused on general changes in attention, i.e., whether a stimulus is fixated or not. Also, most research has employed predominantly cumulative measures of looking (e.g., overall duration of looking at an object, regardless of the location and duration of individual fixations) and used highly-controlled, simple stimuli. Relatively few studies have investigated developmental changes in fixation patterns for more naturalistic and often cluttered visual scenes. In a series of studies Frank and colleagues tracked attention to faces in complex visual scenes across the first 2.5 years of life. They showed that between 3 and 9 months of age infants gradually focus more on faces (Frank et al., 2009), but at a younger age looking is driven more by low-level image salience. This could be attributed partly to the known face “pop-out” effect, where attention is exogenously driven by the presence of a face (Gliga et al., 2009). Over the 1st years, when viewing dynamic displays, infants gradually focus more on selected aspects of faces and bodies—the eyes, the mouth and the hands (Frank et al., 2012). This is likely to provide information on a range of social signals that enable speech comprehension and verbal communication. It remains unknown whether these developmental changes in what infants fixate and for how long in naturalistic scenes lead to the emergence of more complex sequences of fixations, especially when viewing human figures and faces. It is also unclear whether these sequences become increasingly structured, for example, through fixation sequence repetition, when a scene contains human faces, thus leading to more complex, but deterministic patterns of fixation sequences.

Visual scanning is the pattern of eye movements composed of sequences of fixations on different parts of a stimulus (scenes, objects). Novel, dynamic measures of scanning provide information about temporal and spatial characteristics of infant visual scanning behavior. With their use, we can say if there are specific, repeated sequences of fixations or if an infant tended to spend more time looking in greater detail at some area, while sparsely scanning other regions of a scene. In the current study the dynamics of scanning were measured by quantifying fixations that recur in the same location and the repetition of specific re-fixation sequences with the Recurrence Quantification Analysis (RQA; Anderson et al., 2013; Wu et al., 2014). López Pérez et al. (2018) used RQA to quantify differences in global (i.e., re-fixations in previously fixated image areas anywhere in the image) and local (i.e., sequential fixations that consecutively fixate on the same location or specific sequences of fixations or scan paths that repeat) patterns of re-fixations in infant eye-tracking data. They showed that the presence of a face in an array of objects increases both global measures of re-fixations (the overall percentage of recurrent fixations, Recurrence Rate, RR) and the temporal distribution of recurrent fixations (Centre of Recurrence Mass) as well as local measures of re-fixations (the percentage of specific sequences of fixations or scan paths that repeat, Determinism, DET, and the number of sequential fixations that consecutively fixate on the same location (Laminarity, LAM). Higher number of recurrences and repeated fixation patterns in 6-month-olds were also found for naturalistic, cluttered scenes with a person present (Kelly et al., 2019). These results may suggest that domain-specific increases in sensitivity to faces in infancy not only improve face detection in visual displays, but also change the dynamics of scanning of the entire scene. In the presence of a face infants were found to produce fewer, but longer fixations, while in its absence, they were likely to produce a greater number of short fixations, which cover a broader area.

Finally, it remains unknown, to what extent either the domain-general or the domain-specific mechanisms lead to changes in the dynamics of visual scanning across infancy. On the one hand, improvements in oculomotor control allow infants to more efficiently shift their eye gaze, to produce more fixations (e.g., for faster object recognition) or to disengage even from highly attractive stimuli in favor of less attractive stimuli that are related to internal goals. This would suggest that infants who show more rapid orienting or better disengagement are likely to produce more complex patterns of scanning. In other words, individual differences in oculomotor control would be related to dynamic measures of scanning. On the other hand, the domain-specific changes in perceptual sensitivity to faces would lead to age-related changes in the dynamics of scanning for social stimuli only, in comparison with non-social ones.

The present study aimed to (i) investigate the developmental changes in spatial and temporal characteristics of infants' visual scanning (ii) and test whether there are greater changes for displays with social stimuli (i.e., containing human faces and figures) relative to non-social ones. We also aimed to (iii) clarify the role of infant oculomotor skills for the development of visual scanning at 5.5 and 11 months of age. To our knowledge no study to date has tested whether typical development of visual scanning involves changes in the complexity of scanning patterns (i.e., changes in spatio-temporal characteristics) in visual scenes with or without a person present. Thus, we tested this question in a longitudinal design, by using four stimulus categories (social vs. non-social static stimuli and social vs. non-social dynamic stimuli) at two timepoints and applied the dynamic measures of scanning retrieved with the RQA method.

We propose that increased attunement to social stimuli in the second half of the 1st year of life will lead to specific developmental changes in the dynamics of scanning of complex visual scenes if they contain social stimuli. Thus, we predicted an age-related, within-subject increase in re-fixations on faces and more repeated sequences of fixations on faces and body parts (e.g., hands) in social scenes in comparison with scenes without social stimuli. In particular, for static stimuli we expected an age-related increase in DET and LAM measures for social stimuli relative to salient, non-social ones. Also, since faces and body parts increase the rate of re-fixations in an image, we also predicted a similar age-related increase in RR for social relative to non-social static stimuli.

To control for the effects of movement in the stimulus as an exogenous driver of attention, we also compared age-related changes in the scanning of social and non-social dynamic stimuli. For the dynamic stimuli, we hypothesized an increase in RQA parameters for both social and non-social stimuli, because developmental improvements in oculomotor control will facilitate the tracking of moving stimuli across the screen irrespective of their content. However, in line with predictions for static stimuli, we predicted a greater increase in RQA parameters for social than non-social dynamic stimuli.

To investigate the relation between oculomotor control and changes in the scanning of the stimuli, we measured orienting speed at 5.5 months and attention disengagement at 11 months of age with a gap-overlap eye-tracking task. We picked the age of 5.5 months for orienting speed and 11 months for disengagement as the optimal timepoints because of robustness of each skill at a given age (see Hendry et al., 2019). We tested whether individual differences in oculomotor control are associated with age-related changes in RR, LAM and DET for social vs. non-social stimuli in static and dynamic displays. We predicted that the development of oculomotor control will be uniformly associated with RR, DET and LAM changes for all types of stimuli, irrespective of their social content.

## Methods

### Participants

The data were collected in a longitudinal study of attention and cognitive development. A total of 120 infants were recruited at 5–6 months (T1) of age and 94 returned for the second visit at 10–11 months of age (T2). Inclusion criteria for the study were as follows: (a) born between 36 and 42 weeks of gestation and born with a typical birth weight (> 2,500 g), (b) no birth complications or any major medical conditions, and (c) no family risk of autism. All, but two infants were born at term (37–42 weeks). The remaining two infants, who were moderately premature and born at 36 weeks were also included in the final analyses because their eye-tracking data (for both tasks) was within then typical range (1 standard deviation from the group mean). Moreover, recent literature suggests that late prematurity alone is not associated with poorer visual perception after birth (Romeo et al., 2012) or poorer long-term developmental outcomes (e.g., Heinonen et al., 2018), while the effects of prematurity are likely dose-dependent (e.g., Mackay et al., 2010).

At T1 74 participants had complete and usable eye-tracking data for the freeviewing task for all conditions (48 infants had incomplete data for some conditions due to looking away from the screen, excess movement or fatigue). At T2 72 participants had complete and usable data (5 did not complete due to fussiness and further 17 had incomplete data for some conditions). Altogether 44 infants (26 girls, 18 boys) had complete and usable eye-tracking data at both timepoints (T1 and T2) for all task conditions and were analyzed longitudinally. Out of those, 41 infants also had complete data from the gap-overlap task at both timepoints.

The mean age at T1 was M = 164.77 days, range 134–187, while at T2 it was M = 348.09 days, range 332–376. Mean maternal age at infant birth was M = 30.40 years (range 24–39). The sample consisted of predominantly middle-class families from a city with over 1.5 min inhabitants. Maternal education was on average M = 17.29 of completed years (SD = 1.31, range 12–21). Participants included in the final sample did not differ from the excluded ones in terms of participant age at either time point (*t*s < 1.01, *p*s > 0.32) or in terms of maternal age or education (*t*s <0.39, *p*s > 0.70) at T1.

The study was approved by the local institution's ethics committee. All parents gave written informed consent prior to the testing and received a small gift (baby book) and a certificate of their participation.

### Freeviewing Stimuli and Eye-Tracking Task Procedures

Following the warm-up time, infants were seated in a high chair or on a parent's lap ~60 cm from the monitor. The seating was decided by the parent at the beginning of the session. If an infant was seated in a high chair, the parent sat in a chair positioned to the left and just behind the high chair in order to touch and soothe the infant if necessary.

Eye-tracking data were collected on a Tobii T60XL eye-tracker (Tobii Inc.) with a 24 monitor, 60 Hz sampling rate and 0.5° accuracy (value provided by the manufacturer). The five-point infant-friendly calibration involved sequential presentation of a looming colorful stimulus in the centre of the screen and at each corner until the infant fixated it (or until timeout at 2 s). Experimental tasks were presented after the infant successfully calibrated at least 4 points. The freeviewing task was presented in 4 blocks (one picture and one video) mixed with other tasks in a pseudo-random order (not reported here). The duration of the entire eye-tracking session did not exceed 15 min. The stimuli were presented using Matlab Psychophysics Toolbox (Brainard, 1997; Kleiner et al., 2007) and Talk2Tobii package (Deligianni et al., 2011). Infant behavior was monitored and recorded by a remote-controlled CCTV camera for offline control of looking. Except for the monitor and camera lens, the entire area around the eye-tracker monitor was covered with a black cloth to provide a uniform background.

The stimuli consisted of four images, each lasting 10 s (2 social scenes with multiple faces and human figures and 2 non-social scenes with multiple colorful objects, e.g., flowers, easter eggs) and four videos, each lasting 30 s (2 social with portrait view of the human figure saying baby rhymes and 2 non-social cartoon videos with a toy object, a plane or vehicle moving around the screen). Screenshots of sample stimuli are presented in the Supporting Information alongside saliency maps for static stimuli (see Supplementary Figures 1, 3).

### Gap-and-Overlap Task

At both T1 and T2 we used the gap-and-overlap task (Farroni et al., 1999; Elsabbagh et al., 2013) in a version prepared by Wass et al. (2011); for a full description and task validity analyses (see Niedzwiecka et al., 2018). Infants were presented with at least 48 trials (in 4 blocks). An additional block was run until enough usable trials were collected (12 per condition), or 80 trials had been presented or the infant became inattentive. Each trial began with a central target (a cartoon clock, subtending 4.5° visual angle in diameter) appearing after a variable inter-stimulus interval (between 600 and 700 ms in duration). Once the central target was fixated by the participant, a lateral target (a cartoon cloud, subtending 3° in diameter) was presented on either side of the screen 13° away from the centre. There were 3 trial types, presented in equal number in random order: Gap–lateral target appeared 200 ms from the central target offset, Baseline – lateral target appeared as soon as central target disappeared from the screen, Overlap – central target remained on the screen for 200 ms from the onset of lateral target. Saccadic reaction times were measured as the latency between lateral target appearance and the reported position of gaze leaving the central fixation area (a 9° box around the central target). Saccadic latencies lower than 100 and >2,000 ms were excluded. Recent analyses argue for using directly average saccadic reaction times from individual conditions (see Siqueiros Sanchez et al., 2020), hence for each participant we used average Gap saccadic reaction times at T1 as a measure of orienting speed and average Overlap saccadic reaction times at T2 for attention disengagement.

### Eye-Tracking Data Processing

Prior to the RQA analysis, fixation coordinates and durations were extracted using a noise-robust fixation detection algorithm that uses 2K-means clustering (Hessels et al., 2016) and we used most of the suggested default settings for the algorithm. For the Steffen interpolation, we used an interpolation window of 100 ms and an interpolation edge of 2 samples (i.e., 33.33 ms). We chose these values since values longer than 100 ms would lead to interpolation of blinks, which usually take longer than 100 ms (Hessels et al., 2016), while smaller values lead to fewer periods of data loss being interpolated. In the k-means clustering, we applied a sample-by-sample analysis, a clustering window size of 200 ms, down sampling the data (acquisition frequency was 60 Hz) was done using a 8-order Chebysev filter with 0.05 dB ripple in the bandpass to assure that the transitions between fixations are not caused by high-frequency noise in the data at 30, 20, and 15 Hz and a clustering cut-off of 2 times the standard deviation above the k-means weights. Given that fixation durations are typically longer than 150 ms (e.g., Irwin, 1992) the clustering window would contain parts of at most two fixations. Next, after the algorithm estimated all possible fixations, only those that had a minimum duration of 125 ms were considered valid and shorter fixations candidates were excluded. Finally, consecutive fixation candidates close in space and time were merged. To this end, we merged fixation-candidates that were separated in space by <0.7° apart (~13 pixels) and in time by <30 ms. Increasing both parameters would lead to more fixations being merged.

### RQA Analyses

**Figure 1:**
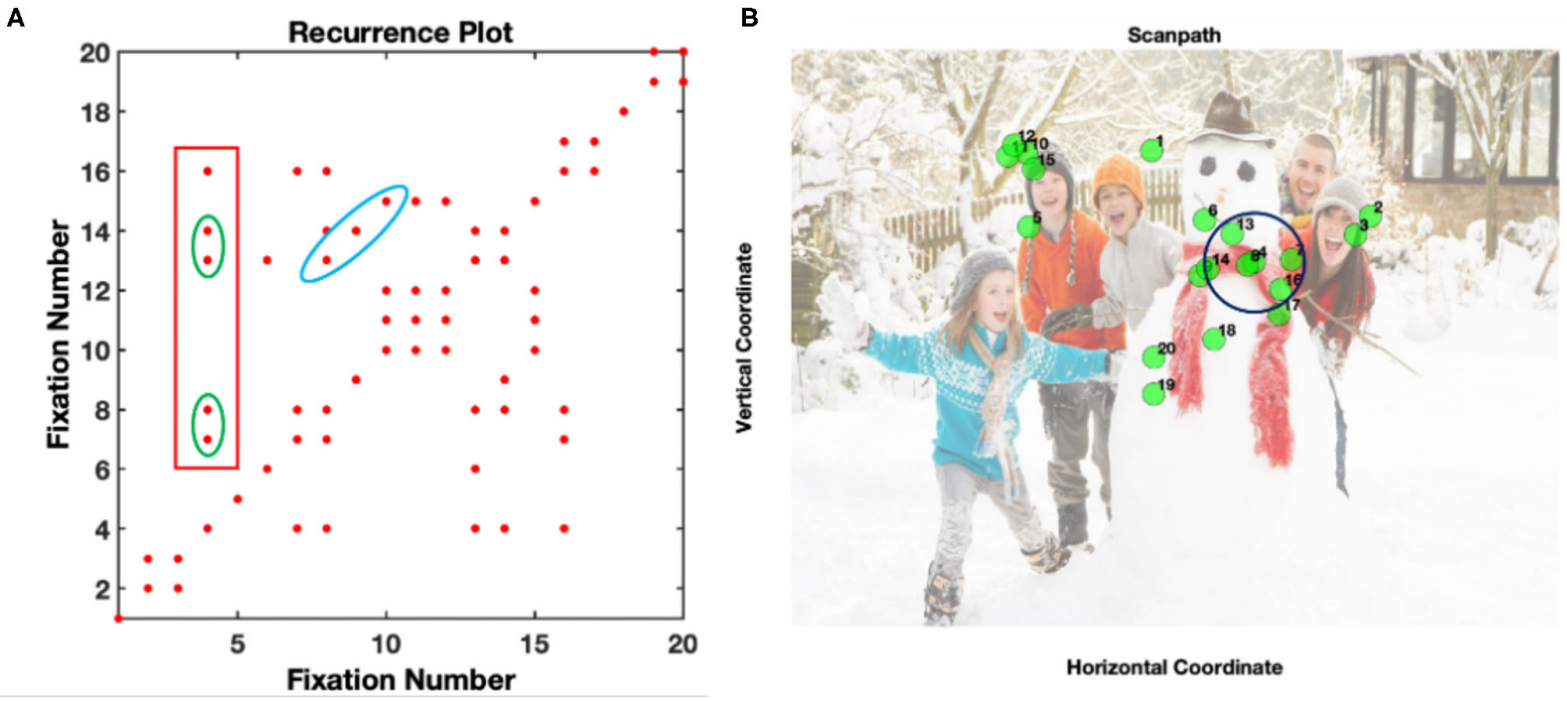
Example of an RQA plot **(A)** and its original fixation coordinates for a social visual scene **(B)**. The blue ellipse represents deterministic fixations sequences where fixations 7, 8, and 9, recurred with fixations 13, 14, and 15 The green ellipse square shows consecutive patterns of fixations (fixations 7-8 and 13-14) at the same location. The red square in **(A)** represents all the fixations that fall within a 64 pixel radius of fixation 4 (see black circle in **B**). This plot comes from the data of one participant.

A trial was included in the analysis if it contained at least 7 fixations, a minimal value necessary to quantify gaze dynamics. This cut-off value allows infants to develop sufficient visual exploration behavior that can be later related to their exploration strategies and increase the possibility of having dynamic patterns of fixations, while saving most of the trials (López Pérez et al., 2021). The dynamics of visual scanning were explored using RQA on fixation sequences in pre-processed (fixation-filtered) eye-tracking data using a Matlab Toolbox found in: http://barlab.psych.ubc.ca/research/ (see detailed description in Anderson et al., 2013). In RQA, fixations are considered recurrent when they fall within a distance radius (e.g., see the radius drawn over fixation 4 in Figure 1B). Consequently, each fixation is compared with all the remaining fixations to construct the RQA plot (Figure 1A). For instance, fixation 4 recurs with fixations 7, 8, 13, 14, and 16 which is represented by a red dot in the plot (Figure 1A). After the reconstruction of the RQA plot, the dynamics of fixations can be quantified with a few parameters. In this paper, we focused on quantifying the global and local patterns of re-fixations and therefore we extracted the following measures:

- RR: represents the percentage of recurrent fixations (i.e., number of re-fixations in previously fixated image areas);
- LAM: percentage of recurrent points that form vertical lines (i.e., sequential fixations that consecutively fixate on the same location). The minimum length of these structures that should be considered was set to two (i.e., two consecutive fixations);
- DET: the percentage of recurrent points that fall on diagonal lines in the RQA plot (i.e., specific sequences of fixations or scan paths that repeat). The minimum length of these structures that should be considered was also set to two.

The number of recurrences (i.e., each red dot) in the RQA plot increases when an area of the image is repeatedly revisited. For instance, if an infant explores the entire scene in detail, then recurrences will be sparser in the plot. These recurrences can be isolated as single points or occur closer together forming different structures. For instance, during the exploration of the scene, there can be a particular fixation pattern that repeats (blue ellipse in 1a). In this case, the particular pattern in fixations 8, 9, and 10 is repeated in fixations 13, 14, and 15. This type of diagonal structure contributes to the DET measure. Likewise, there could be an instant in which a specific, previously fixated area is consecutively fixated, forming vertical or horizontal lines in the plot (fixations 7-8 and 13-14 inside the green square in Figure 1A). These re-fixations at the same location (in the example above it represents the same location as fixation 4) contribute to the LAM.

In this paper, fixations were recurrent if they fell within a 64-pixel radius (e.g., Anderson et al., 2013; López Pérez et al., 2018) of another fixation, which corresponds to a 3.16-degree visual angle. Finally, to account for the variability in FDs in infants we normalized all the RQA measures to consider individual differences (Anderson et al., 2013). This normalization process can be done by redefining the calculation of recurrence to account for FDs as follows:


() $$r_{i,j}=\{\begin{array}{cc} t_{i}+t_{j}, & \;d(f_{i}+f_{j})\leq \rho \\ 0, & \;otherwise \end{array}$$

where fi and fj represent two fixations within a sequence *f i, j* = 1,…, *N*, the *t*_*i*_ and *t*_*j*_ are their associated fixation durations, *d* the distance metric and ρ the radius (see the redefined RQA measures Appendix in Anderson et al., 2013).

### Statistical Analyses

Our main analyses of variance tested whether there are age-related changes (T1 vs. T2) and social vs. non-social differences in RQA measures of scanning. We also tested whether RQA measures changed significantly more for social relative to non-social stimuli. Since visual scanning differs between static and dynamic displays due to movement in the latter producing more exogenous orienting, we opted for analyzing separately static and dynamic conditions with 2 × 2 repeated-measures ANOVAs with social content (social/non-social), and timepoint (T1/T2) for each RQA measure (DET, LAM, RR). Greenhouse-Geisser correction was applied, where necessary. All pairwise comparisons were Bonferroni-corrected. Additionally, we also conducted control analyses on two dependent variables: the number of fixations and average fixation duration with 2 × 2 repeated-measures ANOVAs with social content (social/non-social) and timepoint (T1/T2) as factors (see Supporting Information). These analyses were conducted with SPSS 26.0 package (IBM, Inc.).

Associations between gap-overlap reaction times and change scores for RQA measures were conducted with Pearson correlations. We calculated age difference scores for each of the RQA measures (DET, LAM, RR) in each condition (static/dynamic x social/non-social) for each participant by subtracting T1 values from values at T2 (change score = T2–T1). Thus, positive scores indicated an increase between T1 and T2, while negative scores – a decrease.

Additionally, we conducted exploratory analysis of the line lengths for Determinism measure. Since they were not normally distributed, we used non-parametric test for the within-subject 2 x 2 factorial design [social content (social/non-social), and timepoint (T1/T2)]using R package nparLD (Noguchi et al., 2012; Feys, 2016). Anova-type statistic was calculated for both main effects and the interaction effect.

## Results

### Global Scanning Patterns

#### RR—Static

**Figure 2:**
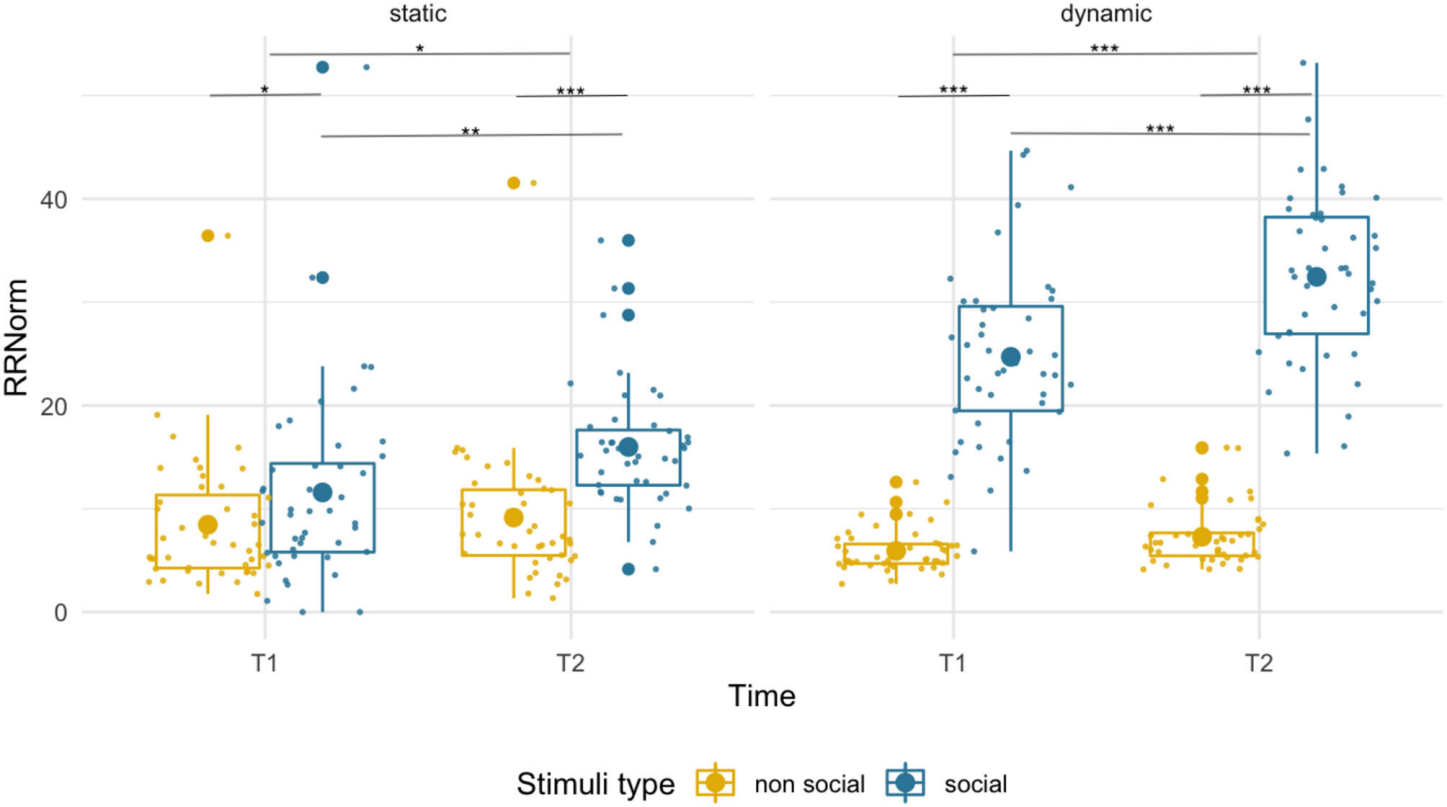
Box plots with individual data showing distribution of normalized recurrence rate for each stimulus type. The big dot inside each box indicates the mean value. Significant differences marked with asterisks: *p < 0.05; **p < 0.01; ***p < 0.001.

We observed an overall higher RR for social than non-social stimuli [*F*_(1,43)_ = 26.73, *p* < 0.001, $\eta_{p}^{2}$ = 0.38] at both T1 (social M = 11.58, SD = 9.37; non-social M = 8.45, SD = 6.18; *p* = 0.042) and at T2 (social M = 15.98, SD = 5.92; non-social M = 9.15, SD = 6.41; *p* < 0.001). While we found a main effect of age [*F*_(1,43)_ = 4.23, *p* = 0.046, $\eta_{p}^{2}$ = 0.09], suggesting higher RR at T2 than T1, the age x condition interaction [*F*_(1,43)_ = 4.81, *p* = 0.034, $\eta_{p}^{2}$ = 0.10] was also significant. Pairwise comparisons revealed an age-related increase only for social stimuli (*p* = 0.009) but not for non-social images (*p* = 0.61). See Figure 2.

#### RR–Dynamic

The recurrence rate was disproportionately higher for social that non-social stimuli [*F*_(1,43)_ = 489.26, *p* < 0.001, $\eta_{p}^{2}$ = 0.92] at both T1 (social M = 24.71, SD = 8.45, non-social M = 5.92, SD = 2.00) and T2 (social M = 32.45, SD = 8.09, non-social M = 7.29, SD = 2.79). Recurrence rate increased with age for both conditions [*F*_(1,43)_ = 30.40, *p* < 0.001, $\eta_{p}^{2}$ = 0.41]. However, we observed a disproportionately higher increase in RR for social than non-social dynamic stimuli [condition x age interaction, *F*_(1,43)_ = 18.15, *p* < 0.001, $\eta_{p}^{2}$ = 0.30].

### Local Scanning Patterns

#### DET–Static

**Figure 3:**
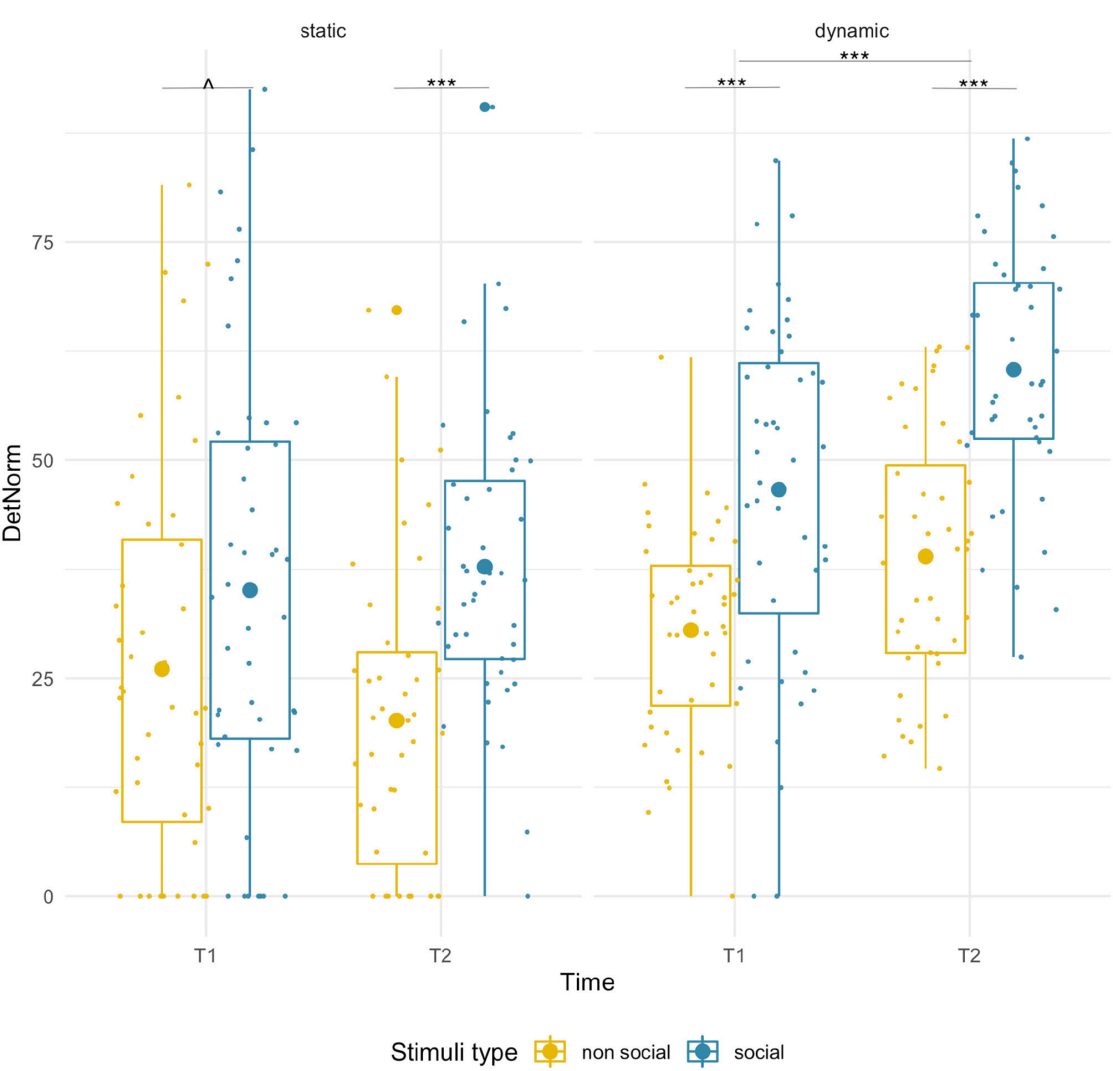
Box plots with individual data showing distribution of normalized determinism for each stimulus type. The big dot inside each box indicates the mean value. Significant differences marked with asterisks: ****p < 0.001; ^∧^ *p* = 0.55.

We found a significant main effect of condition [*F*_(1,43)_ = 21.33, *p* < 0.001, $\eta_{p}^{2}$ = 0.33], which was driven by significantly higher DET for social than non-social stimuli at T2 (social M = 37.78, SD = 16.94; non-social M = 20.16, SD = 17.61; pairwise comparison *p* < 0.001), while for T1 only a near-significant trend in the same direction was found (social M = 35.09, SD = 25.33; non-social M = 26.04, SD = 22.60, *p* =0.055). We did not observe significant age-related changes [*F*_(1,43)_ = 0.28, *p* = 0.60] or age x condition interaction [*F*_(1,43)_ = 1.97, *p* = 0.17]. See Figure 3 and Supplementary Figure 2 for a projection map of the DET onto the sample stimuli.

#### DET–Dynamic

We found age-related increases for both conditions [*F*_(1,43)_ = 31.67, *p* < 0.001, $\eta_{p}^{2}$ = 0.42] and a main effect of stimulus type [*F*_(1,43)_ = 67.62, *p* < 0.001, $\eta_{p}^{2}$ = 0.61]. DET was higher for social than non-social stimuli at both T1 (social M = 46.62, SD = 20.26, non-social M = 30.51, SD = 12.05; *p* < 0.001) and T2 (social M = 60.37, SD = 14.47, non-social M = 38.95, SD = 14.47; *p* < 0.001). No significant interaction was found.

#### LAM–Static

**Figure 4:**
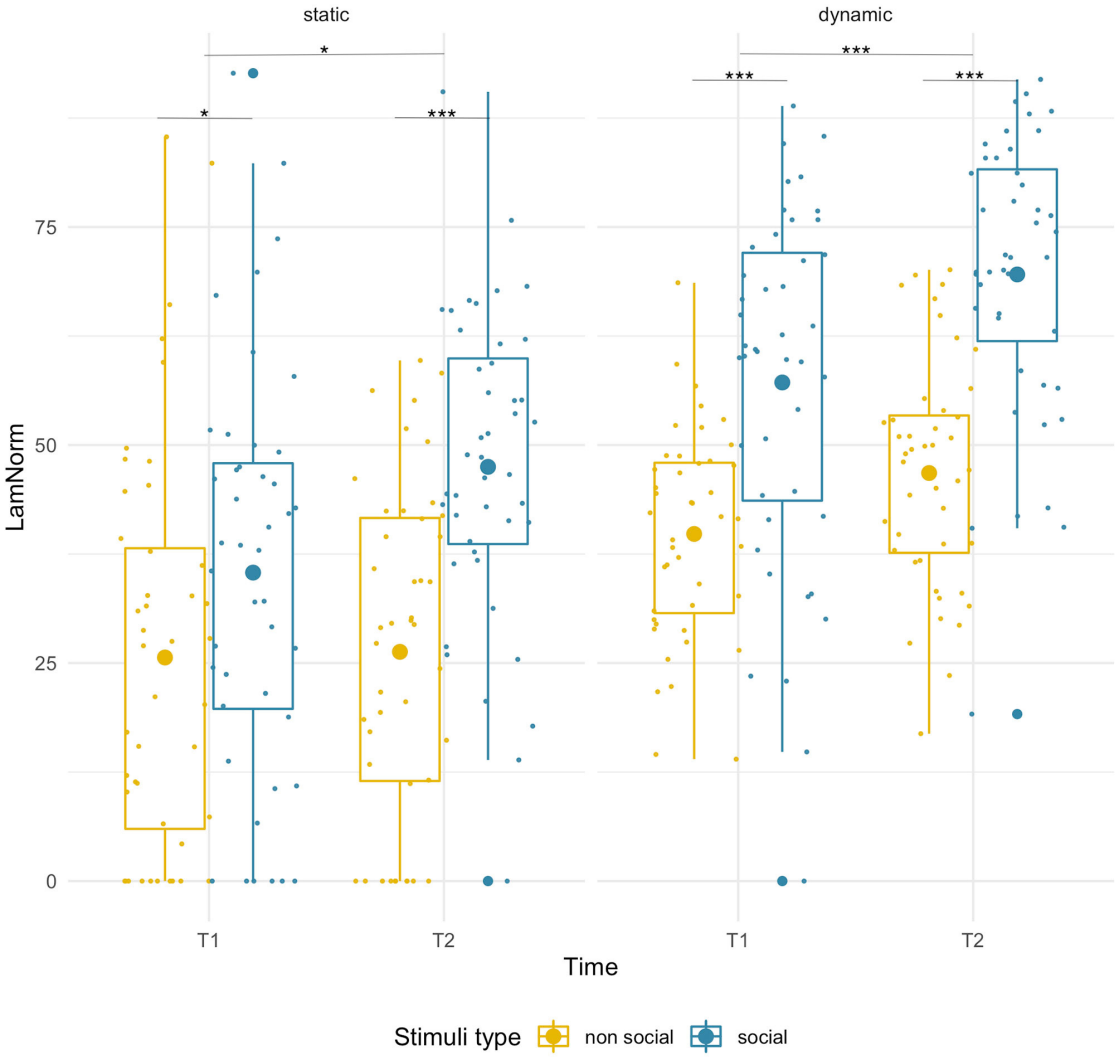
Box plots with individual data showing distribution of normalized laminarity for each stimulus type. The big dot inside each box indicates the mean value. Significant differences marked with asterisks: *p < 0.05; **p < 0.01; ***p < 0.001.

Laminarity was higher for social than non-social stimuli at both timepoints [*F*_(1,43)_ = 26.49, *p* < 0.001, $\eta_{p}^{2}$ = 0.38] and that difference increased with age (T1: social M = 35.37, SD = 22.25, non-social M = 25.64, SD = 23.03; *p* = 0.049; T2: social M = 47.50, SD = 17.58, non-social M =26.29, SD = 18.90, *p* < 0.001). There was a main effect of age [*F*_(1,43)_ = 4.78, *p* = 0.034, $\eta_{p}^{2}$ = 0.10] suggesting higher LAM values at T2, but importantly, we observed a significant age-related increase only for social (T1 vs. T2, *p* = 0.006), but not for non-social stimuli (*p* = 0.89). However, the interaction of factors did not reach significance [*F*_(1,43)_ = 2.80, *p* = 0.102, $\eta_{p}^{2}$ = 0.06]. See Figure 4.

#### LAM–Dynamic

Laminarity was significantly higher for social stimuli than for non-social stimuli [*F*_(1,43)_ = 75.92, *p* = 0.001, $\eta_{p}^{2}$ = 0.64] at both timepoints (T1: social M = 57.18, SD = 20.34, non-social M = 39.80, SD = 11.87, *p* < 0.001; T2 social M = 69.56, SD = 16.07, non-social M = 46.79, SD = 13.05, *p* < 0.001) and there was an age-related increase for both conditions [*F*_(1,43)_ = 26.00, *p* < 0.001, $\eta_{p}^{2}$ = 0.38; pairwise comparisons, both *p*s < 0.005]. No significant interaction was found.

#### DET–Diagonal Line Length (LL) of Fixation Sequences

RQA measures of local scanning are based on lines created by repeated sequences of fixations, either when re-fixating different areas (DET) or scanning one area in more detail (LAM). We found higher DET for social than non-social static stimuli at T2, but not at T1. This means that in social stimuli a greater proportion of re-fixations out of total repeated fixations created sequences (diagonal lines in the RQA plot). A follow-up analysis investigated whether higher DET is explained simply by more repeated sequences (no changes in fixation sequence length) or whether they become longer with age (higher sequence length at T2).

**Figure 5:**
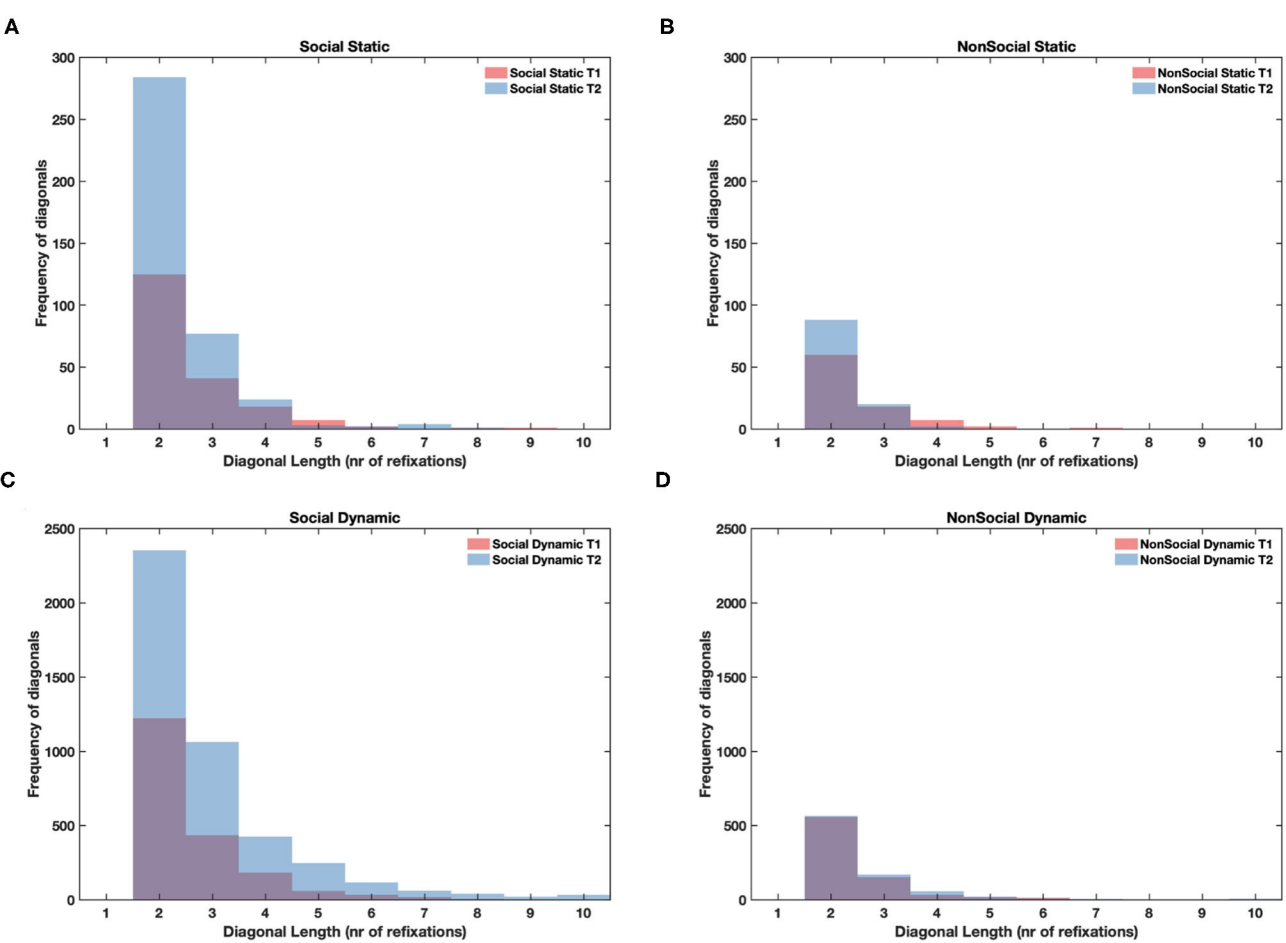
Histograms showing cumulative frequency of diagonal lines of different length (repeated fixation sequences, minimum length of 2 fixations) pooled across all participants for static social **(A)**, static non-social **(B)**, dynamic social **(C)**, and dynamic non-social **(D)** conditions. T1 values are overlaid on top of T2 values, so purple color indicates the overlap of bars between T1 and T2.

We found that in both dynamic and static stimuli the length of repeated fixation sequences was greater for social than non-social stimuli (static: *F* = 15.44, *df* = 1, *p* < 0.001; dynamic: *F* = 27.19, *df* = 1, *p* < 0.001). Moreover, for static stimuli, there was an increase in the line length at T2 relative to T1 only for social stimuli (*F* = 9.07, *df* = 1, *p* < 0.01), but it was not the case for the dynamic ones (*F* = 0.89, *df* = 1, *n.s*.). In dynamic stimuli the line lengths increased between T1 and T2 for both social and non-social stimuli (*F* = 11.29, *df* = 1, *p* < 0.001; main effect of age for static stimuli was not significant *F* = 1.83, *df* = 1, *n.s*.). The mean values are presented in Table 1. These results confirmed that higher determinism of scanning for social than non-social stimuli at T2 involved infants producing longer repeated sequences of fixations when watching faces and bodies regardless of the dynamic/static nature of the display. See Figure 5 for histograms showing non-averaged data of line lengths i.e., taking into account all line lengths across all participants for static and dynamic stimuli.

**Table 1:** Mean and standard deviations for diagonal line lengths in static and dynamic displays at T1 and T2.

**Diagonal line length (DET)**				
	**Static**		**Dynamic**	
	***M*** **(***SD***)**		***M*** **(>***SD***)**	
	**T1**	**T2**	**T1**	**T2**
Social	1.98 (0.90)	2.33 (0.55)	2.40 (0.72)	2.83 (0.47)
Non-social	1.74 (1.18)	1.62 (0.98)	2.34 (0.47)	2.47 (0.55)

*The minimal DET line length parameter was set to 2. However, if the infant did not produce any repeated patterns, the line length was equal to 0. This value was included to compute the mean value for a sample, thus some mean values presented above might be below 2*.

### Control Analyses—Number of Fixations

#### No of Fixations–Static

**Figure 6:**
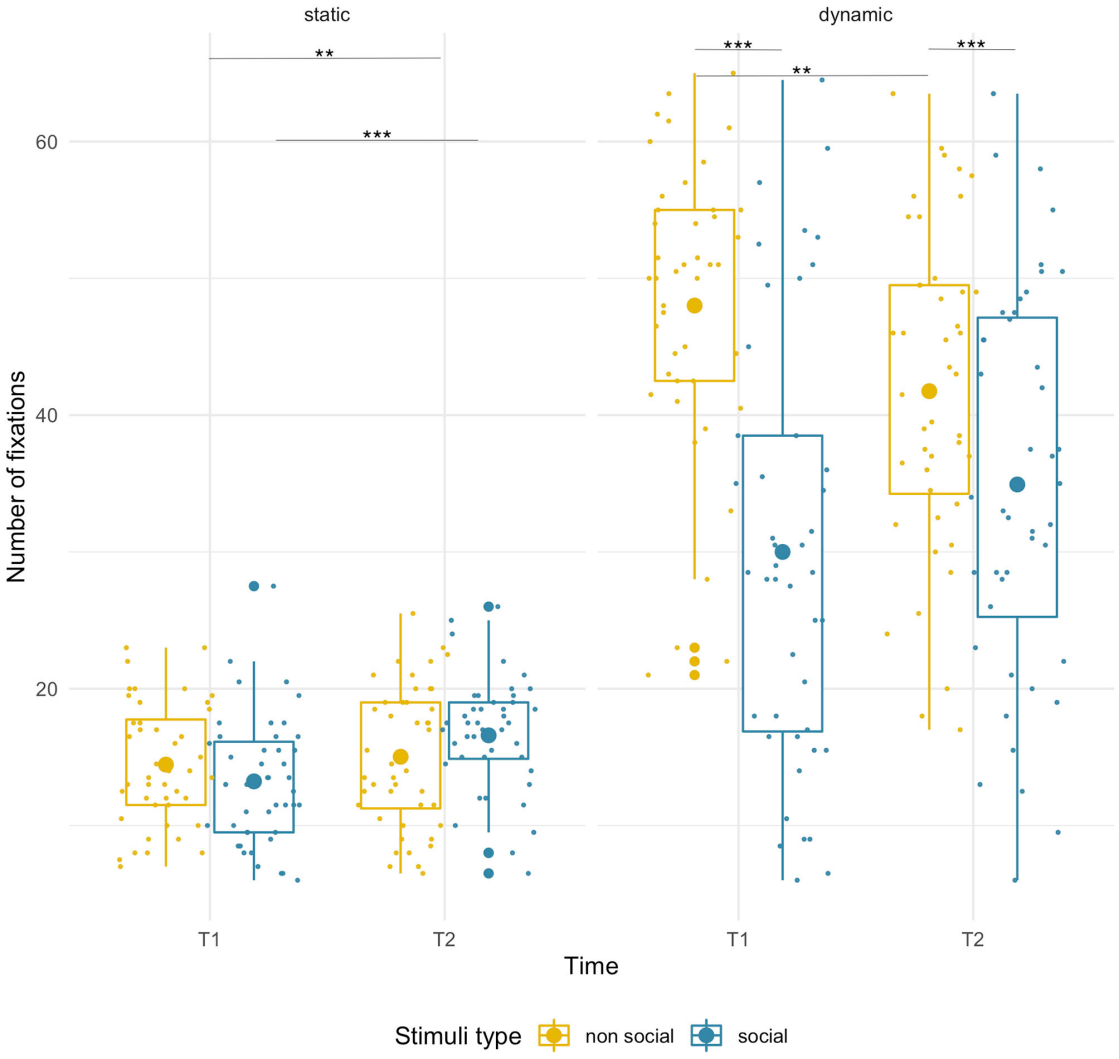
Box plots with individual data showing distribution of the number of fixations for each stimulus type. The big dot inside each box indicates the mean value of number of fixations. Fewer fixations for static stimuli were due to shorter presentation time. Significant differences marked with asterisks: **p < 0.01; ***p < 0.001.

There was a modest, but significant age-related increase [age: *F*_(1,43)_ =7.61, *p* = 0.009, $\eta_{p}^{2}$ = 0.15; condition x age: *F*_(1,43)_ = 7.03, *p* = 0.011, $\eta_{p}^{2}$ = 0.14] in the number of fixations for the social stimuli (T1 M = 13.23, SD = 4.64; T2 M = 16.59, SD = 4.29, *p* < 0.001), but not for the non-social ones (T1 M = 14.46, SD = 4.43; T2 M = 15.02, SD = 5.13, *p* = 0.59). Crucially, the number of fixations did not differ significantly between social and non-social stimuli [*F*_(1,43)_ = 0.09, *p* = 0.76] either at T1 (*p* = 0.084) or at T2 (*p* = 0.07). See Figure 6.

#### No of Fixations–Dynamic

Infants produced more fixations on the non-social than the social dynamic stimuli [main effect of condition, *F*_(1,43)_ = 45.38, *p* < 0.001, $\eta_{p}^{2}$ = 0.51]. However, there were opposite directions of age-related changes for each [condition x age interaction, *F*_(1,43)_ = 12.99, *p* = 0.001, $\eta_{p}^{2}$ = 0.23]. While there was a non-significant trend for the number of fixations to increase for social stimuli (T1 M = 30.00, SD = 15.73; T2 M = 34.93, SD = 14.29; *p* < 0.10), it significantly decreased for non-social videos (T1 M = 48.01, SD = 10.72; T2 M = 41.75, SD = 11.71; *p* = 0.009). No main effect of age was found.

### Individual Differences in Oculomotor Control and Scanning

To better understand how early variation in oculomotor control affects the dynamics of scanning, we explored the associations between RQA measures of scanning and gap-overlap task performance.

#### Orienting Speed

**Figure 7:**
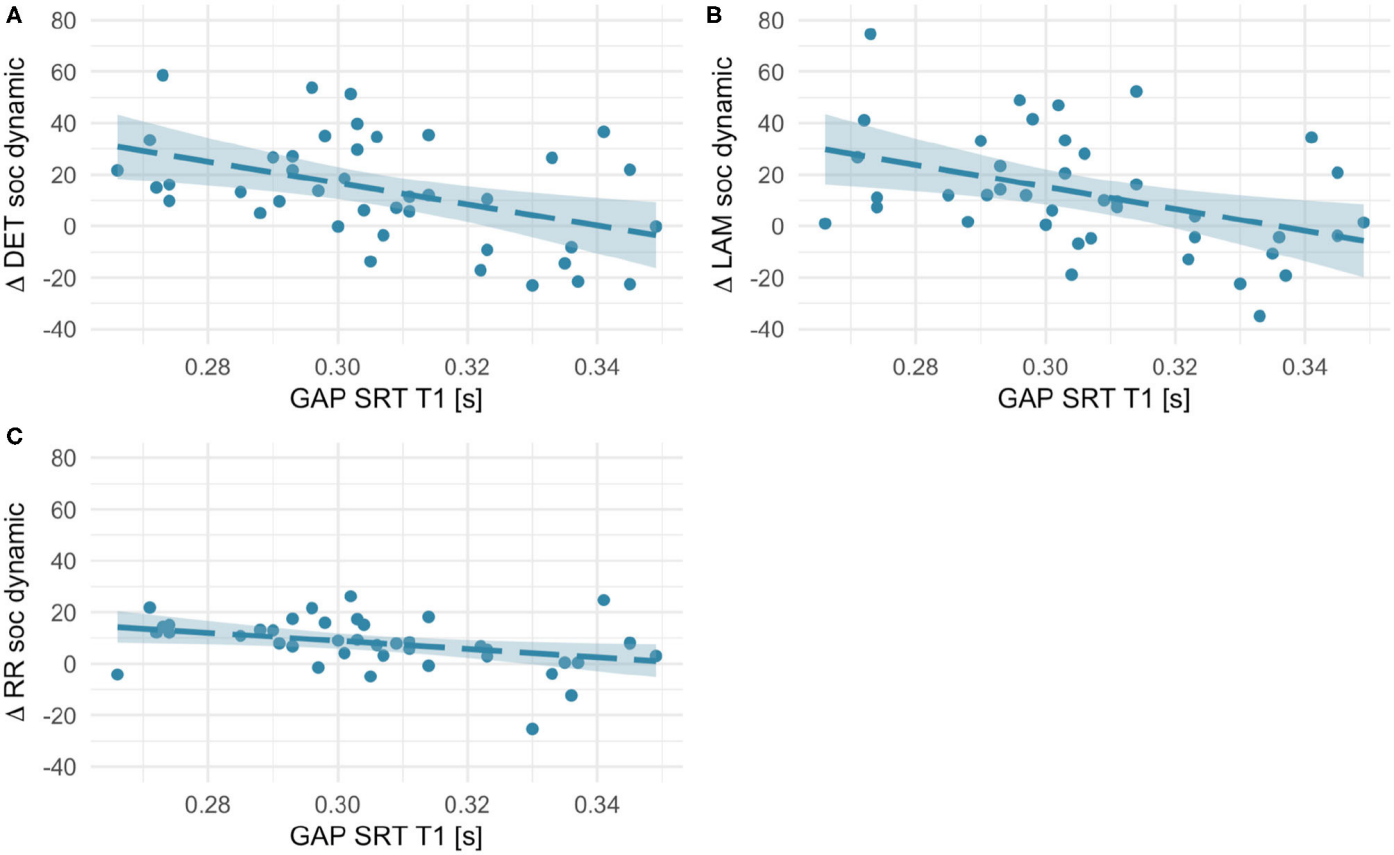
Scatterplots showing associations between orienting speed at T1 (saccadic reaction times in the gap condition) and within-subject age-related change in Determinism **(A)**, Laminarity **(B)**, and Recurrence Rate **(C)** for social dynamic stimuli (T2–T1 values, thus positive values indicate an increase with age and negative values - a decrease). Fit line represents the regression line (with 95% CI).

**Figure 8:**
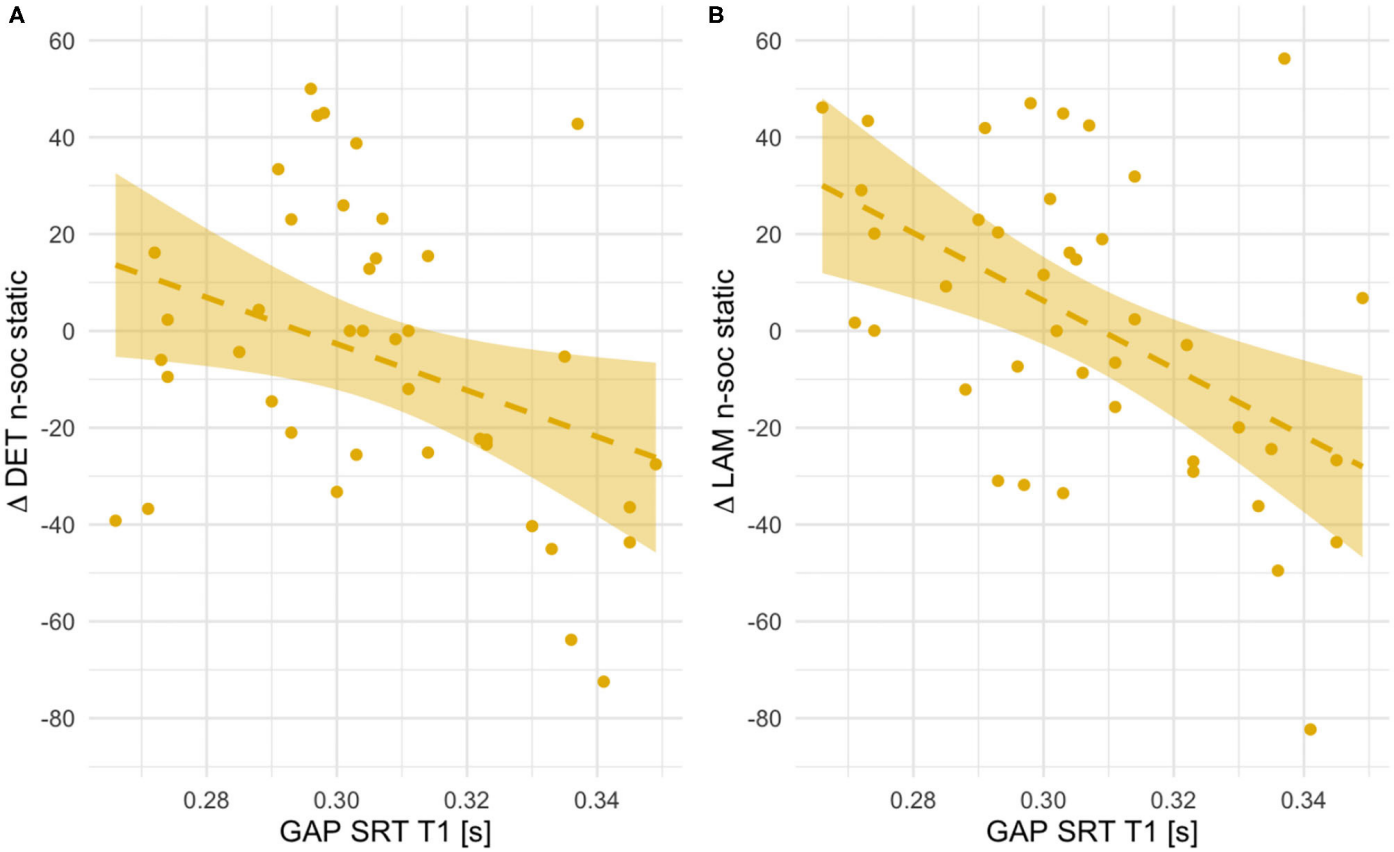
Scatterplots showing associations between orienting speed at T1 (saccadic reaction times in the gap condition) and within-subject age-related change in Determinism **(A)** and Laminarity **(B)** for non-social static stimuli (T2–T1 values, thus positive indicate an increase with age and negative values - a decrease). Fit line represents the regression line (with 95% CI).

First, we investigated whether age-dependent changes in scanning complexity were related to individual differences in orienting speed at T1. We did not find any significant association for social static stimuli (all *r*s < 0.18, *p*s > 0.27). However, RQA change scores for social dynamic stimuli (see Figure 7) significantly negatively correlated with orienting speed at T1 [RR, *r*(41) = −0.36, *p* = 0.021; DET, *r*(41) = −0.45, *p* = 0.003; LAM, *r*(41) = −0.42, *p* = 0.006]. Likewise, we found negative associations with some RQA change scores for non-social static stimuli [DET, *r*(41) = −0.36, *p* = 0.023; LAM, *r*(41) = −0.50, *p* = 0.001; RR, n.s.; see Figure 8]. No significant associations for non-social dynamic stimuli were found (all *r*s < 0.24, *p*s > 0.13).

Only in the case of non-social static images these longitudinal associations could be explained by differences in RR, LAM and DET at T1. Infants with lower orienting speed showed significantly higher number of recurrences [RR, *r*(41) = 0.47, *p* = 0.002], more determinism [DET, *r*(41) = 0.33, *p* = 0.033] and laminarity [LAM, *r*(41) = 0.59, *p* < 0.001]. There were no other concurrent associations at T1 for other types of stimuli. In general, these results suggest that for social dynamic and non-social static stimuli longer saccadic reaction times at 5.5 months are related to smaller increase in re-fixations or in repeated sequences of re-fixations between the two visits.

Attention disengagement skills improve systematically throughout the second half of the 1st year of life. We used overlap SRTs at T2 as a measure of disengagement in our exploratory analysis. We did not find any significant associations of overlap SRTs with change scores for social static (all *r*s < 0.18, *p*s > 0.25), social dynamic (all *r*s < 0.21, *p*s > 0.17), non-social static (all *r*s < 0.17, *p*s > 0.27) or non-social dynamic stimuli (all *r*s < 0.20, *p*s > 0.20; see Supplementary Table 1 for further details).

## Discussion

Although infants preferentially attend to faces from an early age, both their domain-specific (face processing) and domain-general (oculomotor) skills undergo considerable improvements over the 1st year of life. The effects of those emerging skills on the development of visual scanning and structuring of fixations when viewing naturalistic, complex images are largely unknown. We investigated longitudinally the developmental changes in the dynamics of visual scanning in infancy and tested whether they are greater for social than non-social stimuli. To control for the effects of movement in an image we analyzed both static and dynamic stimuli. We also investigated the role of individual differences in infants' oculomotor skills for these changes in scanning.

Our results indicate that scanning of naturalistic scenes containing people changes considerably between 5.5 and 11 months of life. Infants produced significantly more re-fixations in previously fixated areas, they also repeated entire sequences of fixations, while the sequences became longer for social in comparison with non-social scenes. Finally, we found that infants' individual oculomotor skills were not associated with age-related changes in the scanning of static social scenes, while some associations for dynamic social scenes were found. Altogether our study indicates the presence of domain-specific changes in the scanning of social stimuli between 5.5 and 11 months of age, which is unlikely explained by developmental gains in oculomotor control.

We captured complex, dynamic patterns of fixations by using a recently developed method, the Recurrence Quantification Analysis (RQA), which quantifies the global and local patterns of repeated fixation sequences without being distorted by variation in temporal and spatial properties of individual fixations. Our main finding is that between 5.5 and 11 months of age both global and local structuring of fixations in visual scenes with people change considerably in comparison with non-social scenes. Recurrence Rate (RR) derived from RQA measures the global (across the entire image) proportion of re-fixations in previously fixated locations. For crowded pictures containing multiple people we found a within-subject age increase in the RR. For similarly crowded non-social images the RR remained constant at both ages. We obtained similar results for dynamic stimuli–a significantly higher increase in RR between timepoints for videos of a talking face in comparison to videos of moving toys.

Measures of Laminarity (LAM) and Determinism (DET) capture locally-repeated patterns of fixations, which occur only in some areas of an image. Thus, LAM and DET are particularly useful for understanding the effect that the presence of faces and hands have on the tendency to produce specific fixation sequences. We found higher LAM and DET for social stimuli, both static and dynamic, in comparison to non-social ones. Higher Laminarity in social images means a tendency to produce consecutive fixations in selected locations of an image (mostly faces, when present, see López Pérez et al., 2018, 2021), which are explored in more detail relative to scanning of non-social images. Determinism quantifies the repetition of exact same fixation sequences or scanpaths later in a trial and we found higher DET in social than non-social stimuli, both static and dynamic. This means that infants produced more repeated sequences of fixations when a scene contained people in comparison with scenes containing non-social objects. More specifically, for static images we observed that the disparity in DET between social and non-social images did increase with age, although we did not find an overall significant increase in DET for static social scenes. To explain this result, we conducted a follow-up analysis of DET line lengths, which tested for age-related changes in the average number of fixations in a repeated sequence. These repeated sequences became significantly longer with age only for static social stimuli but not for control non-social stimuli.

Our results add several new findings to the literature on the development of visual scanning of social stimuli. First, they suggest a selective, age-related increase in the complexity of scanning for scenes containing humans. It involves both a global increase in the proportion of recurring fixations, as well as an increase in the length of repeated patterns of fixations. These results are consistent with Kelly et al. (2019), who showed higher LAM, DET and RR in infants for static scenes with a human figure in a cross-sectional design. Second, we show in a longitudinal design that this selective increase in complexity for social static stimuli occurs consistently within individuals across the second half of the 1st year of life.

We interpret our results as evidence for domain-specific, selective fine-tuning of visual scanning for social stimuli in infancy. We observed a selective age-related increase in re-fixations for static and dynamic social scenes, and an increase in the length of repeated sequences of consecutive fixations in social scenes. We propose that this process is likely related to perceptual narrowing for human faces and figures that occurs in the second half of the 1st year of life (e.g., Wheeler et al., 2011), although cultural influences on scanning are also likely (Haensel et al., 2020). Perceptual narrowing involves a specialization of perceptual systems so that they become highly sensitive only to faces that commonly occur in an infant's environment, likely supporting learning from socially-relevant adults (e.g., those that speak native language, Begus et al., 2016). One of the functions of visual scanning is to rapidly detect and orient toward social partners in order to establish interactions involving e.g., toy manipulation and attention shifting (see e.g., Abney et al., 2020). More efficient scanning of socially-relevant objects, e.g., by fixating only faces and hands (Frank et al., 2012) is likely to facilitate this process. Our results highlight the exact process of fine-tuning of visual scanning, which supports more efficient attention to social partners and their actions. We demonstrate that when faced with displays containing human figures infants do not simply fixate more on faces or body parts, but that their scanning becomes more organized and complex. They produce more repetitions of fixation sequences when viewing social images, while those sequences also become increasingly longer with age. Altogether, between 5.5 and 11 months of age visual scanning of scenes with social content becomes more complex and structured in comparison with non-social scenes.

We also explored whether developmental changes in the complexity of scanning patterns depend on individual differences in early oculomotor skills. Importantly, our exploratory analyses failed to show any association between age-related increases in the recurrence or sequencing of fixations for static social stimuli and early differences in oculomotor control. No correlations of DET, LAM or RR were found either with orienting speed at 5.5 months or attention disengagement at 11 months. This may suggest that the fine-tuning of visual scanning for static social stimuli progresses relatively independently of variation in infants' domain-general, oculomotor skills. These results seem consistent with those obtained by Hunnius et al. (2006) with 6.5-month-old infants scanning dynamic videos of talking face. They found no positive association between the development of scanning dynamic social stimuli and attention disengagement skill. Our finding stands to some extent in contrast with the results of Frank et al. (2014), who found greater duration of looking at faces for infants with shorter reaction times in a visual search task. However, these disparities could be attributed to task differences as visual search tasks place considerably higher demands on attentional resources than a simple orienting task used in our study. Kelly et al. (2019) did not find associations between face detection in static displays and measures of oculomotor control. We also highlight the fact that our study investigated changes in the patterning of fixations, while the number and duration of fixations were shown to be comparable across the two static conditions. Thus, the associations previously found for the overall duration of looking may not necessarily translate onto differences in the structuring of fixation sequences. Altogether, our results suggest that early domain-general oculomotor skills are not related to developmental increases in the complexity of scanning for static social stimuli.

For dynamic stimuli, the pattern of results was more complex. We found a significant longitudinal association between orienting speed at 5.5 months of age and both global and local scanning of talking faces. Infants that were faster to orient to a peripheral target showed greater increases in RR, LAM and DET for dynamic social stimuli. No comparable associations were found for moving non-social objects. Thus, more rapid attention shifting at 5.5 months may facilitate the emergence of longer and repeated sequences of fixations on talking faces later on. Taking together the results for static and dynamic social stimuli, one ought to consider the differences in task demands. Static stimuli, although crowded and rich in salient objects, were nonetheless presented for a shorter duration and did not contain an auditory track. On the other hand, our dynamic social stimuli were faces speaking baby rhymes in native language, thus presenting much higher demands on both attention to articulation and speech comprehension, thus differentiating infants on the basis of their orienting speed. It is likely that the presence of articulation and speech may account for the association with individual differences in orienting speed. This interpretation is supported by the fact that no comparable association was found for dynamic non-social stimuli, where moving objects were accompanied by irrelevant background music. In summary, it is likely that changes in the complexity of scanning for dynamic social stimuli are related to the presence of facial movement and speech rather than the mere presence of facial configuration in a display.

### Limitations

Finally, we discuss some aspects of the RQA methodology and study limitations. One criticism of RQA measures in eye-tracking is that they can be distorted by differences in low-level fixation parameters, such as the number of fixations and require a certain minimal number of fixations per trial to be meaningful. We have minimized the effects of these factors by controlling for variation in average fixation durations through normalization of RQA measures (see full explanation in López Pérez et al., 2018) and by excluding the participants that did not produce a sufficient number of fixations (at least 7). Also, higher complexity of scanning for social relative to non-social stimuli cannot be explained by the number of fixations. Although the latter increased with age in static stimuli, there were no significant differences between social and non-social stimuli at T2. For dynamic stimuli we observed more fixations for non-social than social displays. These results further highlight the fact that the development of attention to social stimuli involves changes in the structuring of fixation sequences, rather than mere changes in the quantity or duration of fixations.

We note that the conclusions of our study are somewhat limited because the static and dynamic stimuli could not be compared directly due to large differences in presentation time and in low-level visual properties. Although it is extremely difficult to create perfectly comparable naturalistic static and dynamic displays that are sufficiently engaging for infants, our results warrant further research with better controlled displays that vary only in social content. We also acknowledge that the RQA measures of fixation recurrences do not take into account the movement of the object itself in dynamic displays, thus our results for the dynamic stimuli should be interpreted with some caution. Although we tried to select dynamic images with reduced screen movement, there is a need to develop RQA measures of fixation dynamics that robustly control for the amount of object movement in videos.

### Implications

In recent years the research on infant visual scanning has focused on the mechanisms that drive attention to social partners not only in simple experimental settings, but also under more naturalistic conditions. In our study we found some differences in the development of scanning of static vs. dynamic displays. Our results may help to develop dynamic measures of scanning for naturalistic studies of looking during real-life social interactions, e.g., using head-mounted eye-tracking. They may also help to understand the sources of variability in early social attention under naturalistic settings. This is particularly important given the fact that RQA methods are proving useful for quantifying early individual differences in visual scanning of complex displays, which have predictive value for later communicative skills (López Pérez et al., 2021).

Finally, our results may suggest a new line of research on the optimisation of visual scanning of complex social stimuli in early development. Attending to multiple elements of a social interaction is highly challenging and demanding for infants, so the emergence of deterministic patterns of scanning may help to achieve greater attentional efficiency. Our data shows that it is achieved not only by increasing selectivity on some areas of an image (e.g., eyes, mouth or hands), but also through a stabilization of more complex patterns where entire sequences of fixations are repeated multiple times. This idea seems particularly relevant to research on emerging atypicalities in social attention, where high variability and low efficiency of scanning are often key features (see e.g., Shic et al., 2011). However, since atypical scanning of social stimuli likely emerges in the 2nd year of life (Bedford et al., 2012), more research is needed to understand the typical dynamics of scanning in this subsequent period of life.

## Conclusions

Our study for the first time found selective developmental changes between 5.5 and 11 months of age in the structuring (repetition) of fixation sequences and the overall rate of recurring fixations for static images containing human figures. These changes were not related to infants' individual skills in eye movement control. We interpret our findings as evidence for the process of domain-specific fine-tuning of attentional systems to more efficiently scan social stimuli in infancy.

## Data Availability Statement

The raw data supporting the conclusions of this article will be made available by the authors, without undue reservation.

## Ethics Statement

The studies involving human participants were reviewed and approved by the longitudinal study received ethics clearance from the Local Committee at the Faculty of Psychology, University of Warsaw, Poland. Written informed consent to participate in this study was provided by the participants' legal guardian/next of kin. The individual(s) provided their written informed consent for the publication of any identifiable images or data presented in this article/Supplementary Material.

## Author Contributions

PT designed the study and oversaw its execution, secured the funding, processed data for the additional eye-tracking task (gap-overlap), conducted main statistical analyses, wrote the first draft, and edited subsequent versions of the manuscript. DLP ran the RQA analysis and pre-processing of eye-tracking data for the main task and edited different versions of the manuscript. AR conducted statistical analyses, visualized data, and edited different versions of the manuscript. AM-K managed and conducted the data collection and edited the manuscript. All authors contributed to the article and approved the submitted version.

## Funding

The data collection was funded by a Polish National Science Centre grant (2011/03/D/HS6/05655, PT) and additionally supported by the People Programme (Marie Curie Actions) of the EU FP7 (Grant no. PCIG10-GA-2011-304255, PT). The data analysis was supported by the European Union's Horizon 2020 research and innovation programme under the Marie Skłodowska-Curie grant agreements #642996 (BRAINVIEW) and #814302 (SAPIENS) and by the Institute of Psychology, Polish Academy of Sciences.

## Conflict of Interest

The authors declare that the research was conducted in the absence of any commercial or financial relationships that could be construed as a potential conflict of interest.

## Publisher's Note

All claims expressed in this article are solely those of the authors and do not necessarily represent those of their affiliated organizations, or those of the publisher, the editors and the reviewers. Any product that may be evaluated in this article, or claim that may be made by its manufacturer, is not guaranteed or endorsed by the publisher.
