# Supplementary material for: Selective Changes in Complexity of Visual Scanning for Social Stimuli in Infancy
Source: Front Psychol. 2021 Nov 2;12:705600. doi: 10.3389/fpsyg.2021.705600 (PMC8593402; doi:10.3389/fpsyg.2021.705600)
Supplement: Supplementary file 1 [file Data_Sheet_1.pdf]

Selective changes in complexity of visual scanning for social stimuli in infancy

Supporting Information

Supporting Methods

1. Examples of stimuli

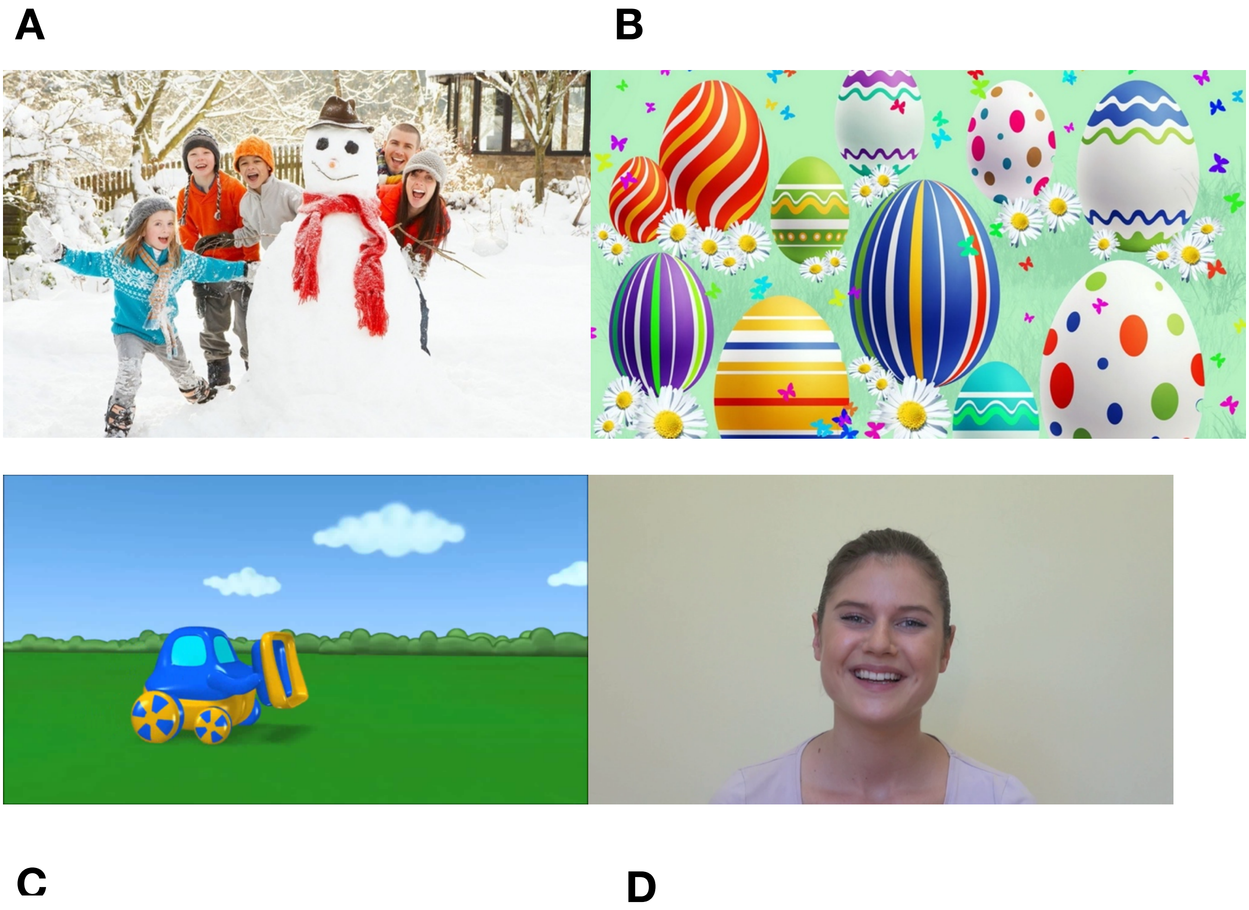

Figure S1. Examples of stimuli used at both timepoints: social static image (A), non-social static image (B), non-social dynamic video (C) and social dynamic video (D).

2. Heat Maps - back projection of Determinism

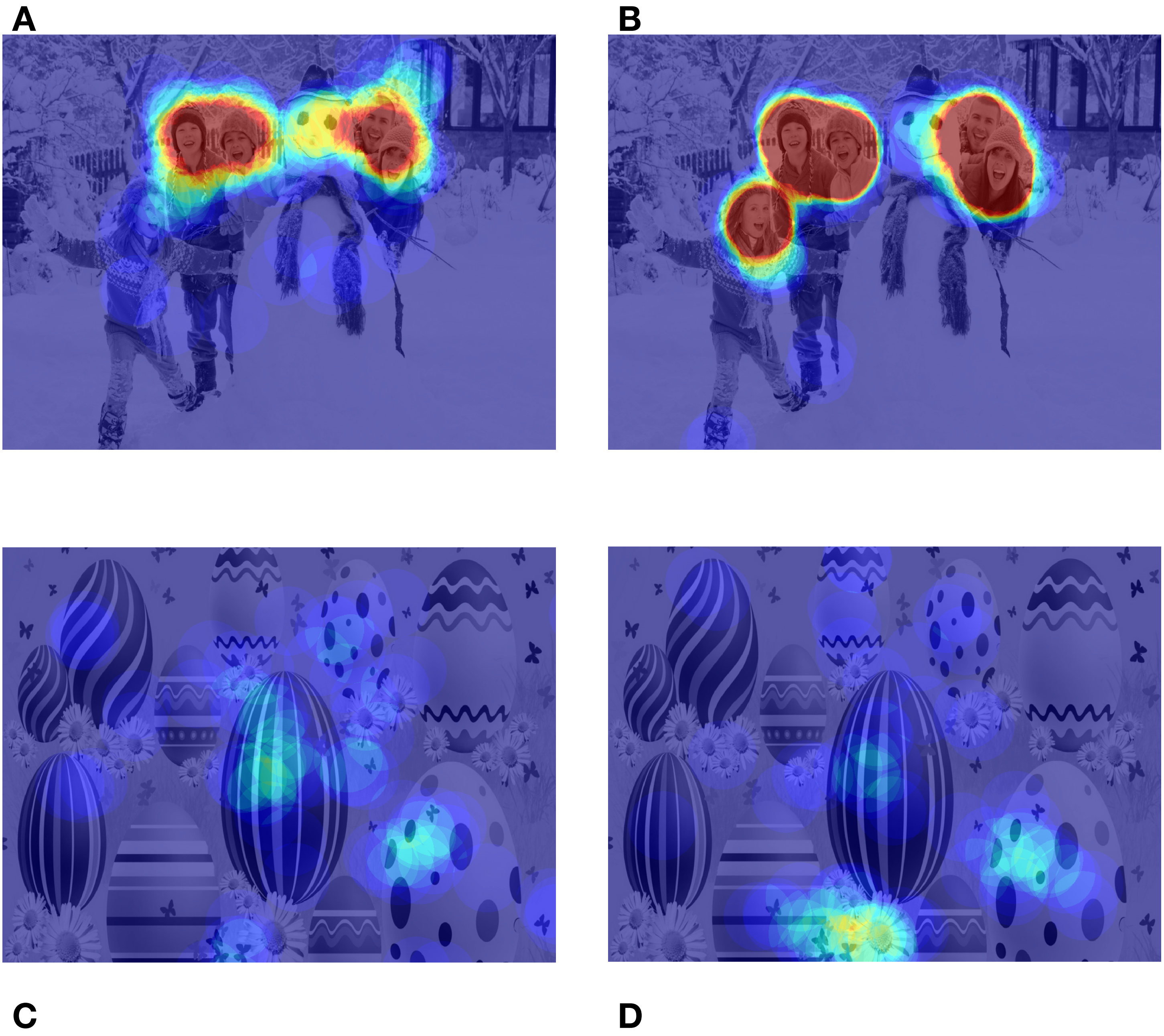

Figure S2. Heatmaps back projecting Determinism onto the image space for a sample stimulus in the static social T1 (A), static social T2 (B), static non-social T1 (C) and static non-social T2 (D) condition. Images present the results averaged across participants. For information on the method for back projection see López Pérez, D., Radkowska, A., Raczaszek-Leonardi, J., Tomalski, P., & The Talby Study Team (2018). Beyond fixation durations: Recurrence quantification analysis reveals spatiotemporal dynamics of infant visual scanning. Journal of vision, 18*(13), 5. https://doi.org/10.1167/18.13.5*

3. Saliency Maps

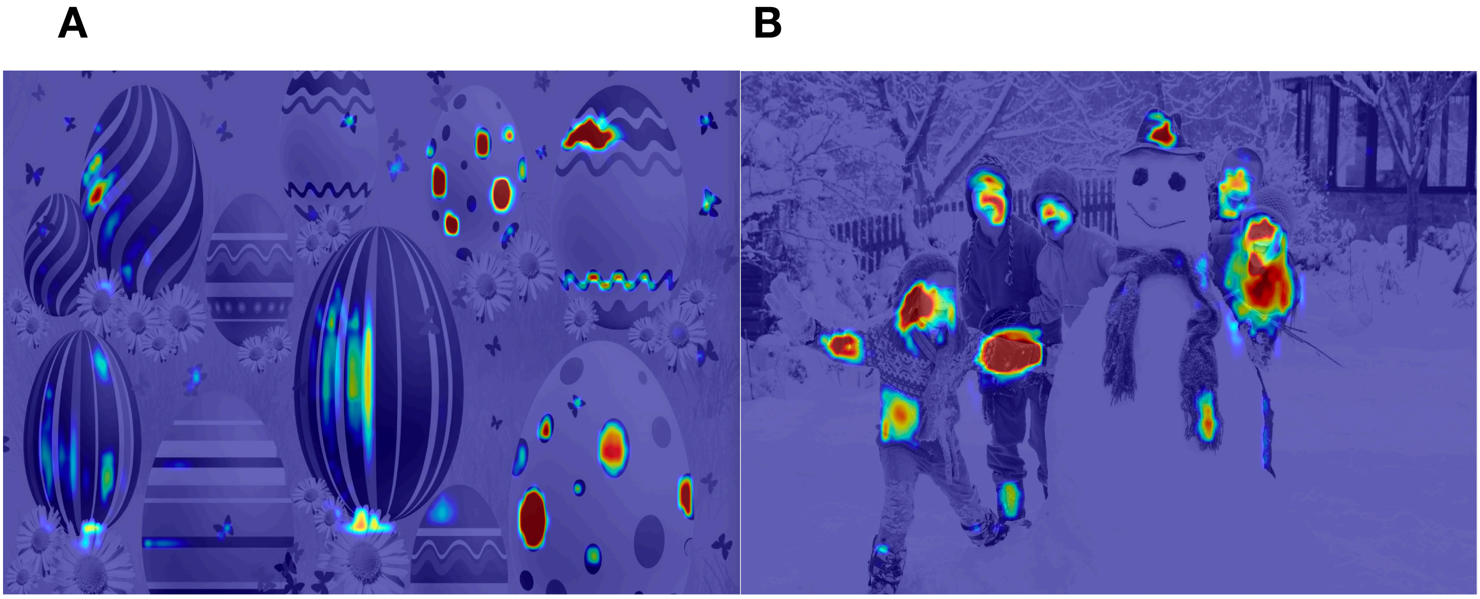

Figure S3. Saliency map for a sample stimulus in the static non-social (A) static social (B) condition. Saliency measure based on Walther D. and Koch C. (2006), Modeling attention to salient proto-objects. Neural Networks 19, 1395-1407.

Supporting Results

1. Control analyses for average fixation duration T1 vs T2

Average fixation durations - static. There were no significant differences in the average duration of fixation between conditions and timepoints. 2 x 2 ANOVA show no significant effects or interactions.
Average fixation duration - dynamic. There were no significant differences between conditions at either timepoint ($F(1,43) = 0.03$, $p = .86$), but fixations were on average longer at T2 than T1 for both social and nonsocial videos (main effect of age, $F(1,43) = 9.35$, $p = .004$, $\eta_p^2 = .18$).

2. Gap-overlap descriptive data

At T1, infants included in the final analyses contributed on average 11.29 valid trials in the Baseline condition (SD = 4.43), 11.46 in the Gap condition (SD = 4.14), and 11.32 valid trials in the Overlap condition (SD = 4.93). Mean Gap SRT was equal to 306.64 ms (SD = 22.4), and mean Overlap SRT equaled to 464.85 ms (SD = 90.0).

At T2, infants included in the analyses completed on average 12.52 valid trials in the Baseline condition (SD = 4.18), 12.00 in the Gap condition (SD = 4.13) and 13.29 valid trials in the Overlap condition (SD = 4.27). Mean Gap SRT was equal to 290.0 ms (SD = 26.45), and mean Overlap SRT equaled to 452.4 ms (SD = 61.4).

3. Full correlation table of gap SRTs at T1 and overlap SRTs at T2 with T2-T1 change scores for RQA measures (*N* = 41).

Table S1. Correlations between change scores (T1-T2) in RQA measures (RR, DET, LAM) and saccadic reaction times in gap condition at T1 and overlap condition at T2. Negative correlations mean shorter saccadic reaction time being related to increase in Recurrence Rate, Determinism or Laminarity between visits.

		**Gap SRT T1**	**Overlap SRT T2**
	**RQA change score**		
**Static social**	Δ RR	*r* = .07, *p* = .65	*r* = .14, *p* = .39
	Δ DET	*r* = .10, *p* = .53	*r* =-.006, *p* = .97
	Δ LAM	*r* = .18, *p* = .27	*r* = .11, *p* = .49
**Static non-social**	Δ RR	*r* = -.27, *p* = .08	*r* = .07, *p* = .68
	Δ DET	***r* = -36, *p* = .02**	*r* = .18, *p* = .27
	Δ LAM	***r* = -.50, *p* = .001**	*r* = .15, *p* = .35
**Dynamic social**	Δ RR	***r* = -.36, *p* = .02**	*r* = -.05, *p* = .74
	Δ DET	***r* = -.45, *p* = .003**	*r* = -.21, *p* = .19
	Δ LAM	***r* = -.42, *p* = .006**	*r* = -.12, *p* = .47
**Dynamic non-social**	Δ RR	*r* = -.24, *p* = .13	*r* = .13, *p* = .41
	Δ DET	*r* = -.09, *p* = .57	*r* = -.20, *p* = .20
	Δ LAM	*r* = -.23, *p* = .14	*r* = -.05, *p* = .74
